# Modeling the spatial and seasonal distribution of offshore recreational vessels in the southeast United States

**DOI:** 10.1371/journal.pone.0208126

**Published:** 2018-11-28

**Authors:** Nancy Montes, Robert Swett, Robert Ahrens

**Affiliations:** 1 Florida Sea Grant College Program, University of Florida, Gainesville, Gainesville, Florida, United States of America; 2 Fisheries and Aquatic Sciences Program, School of Forest Resources and Conservation, University of Florida, Gainesville, Florida, United States of America; Aristotle University of Thessaloniki, GREECE

## Abstract

Understanding the distribution and intensity of recreational boating activities is key for managing safety as well as environmental and social impacts. Recreational boating is a very important component of the diverse maritime traffic in the southeastern United States. The seasonal distribution of offshore recreational vessels in waters off the coast of Northeast Florida and Southeast Georgia was modeled using several techniques (Poisson, negative binomial, hurdle and zero inflated modes, generalized additive models, and generalized mixed models) and by combining map-based information provided by recreational boaters with environmental and geographical variables to find the most parsimonious model. Based on model performance, the final model analysis was conducted using a GAM approach with a negative binomial distribution. The best seasonal models explained between 86.1%– 88.6% of the total deviance. For most seasons, a model that included latitude, longitude, interaction between latitude and longitude, chlorophyll *a* concentration, and abundance of artificial reefs resulted in the best fit. The only exception was the model for the summer season, which did not include chlorophyll *a* concentration. Given the complexity of the study area, with a number of maritime activities and several marine species co-occurring, these models could provide information to analyze the distribution and overlap of recreational boating trips with other maritime activities (e.g., cargo ships, commercial vessels) and species (e.g., right whales, sea turtles, sharks). These analyses could be used to decrease harmful interactions among these groups and activities.

## Introduction

The southeastern United States is a complex region where diverse maritime activities occur. In 2012 there were almost two million people inhabiting the seven coastal counties that comprise the study area in northeast Florida and southeast Georgia [1]. In addition, almost 87,500 recreational boats were registered in these seven counties [2,3]. This represents an average of one boat for every twenty-two residents, suggesting that recreational boating is an important and popular activity in the study area. Montes and others [4] estimated that recreational vessels constitute 86% of vessel traffic at the main inlets of the study area. This includes boaters from nearby counties, and from several states of the USA and other countries (e.g., Canada). However, information about the spatial and temporal distribution of offshore recreational vessel traffic and activity in the study area is limited [4,5].

Understanding the distribution and intensity of recreational boating activities is key for managing safety, environmental, and social impacts [6]. Managers have made use of vessel traffic studies to address a number of issues, including human safety, waterway maintenance, channel marking, user conflicts, wildlife management, and allocation of infrastructure and boating facilities. For instance, Wu and others [7] examined boating safety issues and found that, in general, the relative incident rate (e.g., number of accidents) increased as weather conditions (e.g., wave height, sea surface temperature, air temperature, ice concentration, fog presence, and precipitation) deteriorated. Swett and others [8] designed a GIS-based decision support system that considers the physical conditions and boating characteristics of the Intracoastal Waterway (ICW) when assessing the need for boating safety zones. Gorzelany [9] documented the effectiveness of regulatory speed zones in Miami-Dade and Palm Beach counties in Florida and found that compliance with speed zones is significantly related to vessel size and type. Bauduin and others [10] compared the spatial distribution and overlap of recreational vessels and manatees in Collier County, Florida. Their analyses provided a map showing areas with high/low probability of manatee/recreational vessel co-occurrence. Information from a regional waterway management system for Manatee, Sarasota, Charlotte, and Lee counties in Florida provides a tool and decision options for managing navigation channels in those counties [11].

Several approaches for analyzing spatial patterns of recreational boaters have been utilized. These include geostatistics and kernel density analysis [9,12,13,14,15,16], vessel traffic simulations using agent-based models [17,18,19], and distribution models based on external (e.g., behavioral, geographic, and/or networks) and situational (e.g., weather, water temperature, time, season) variables [20,21,22]. Nevertheless, the effect of external variables on the distribution of recreational boaters and the relationship among the different variables is not well understood (especially at a seasonal scale and within the marine environment). Furthermore, there is an array of statistical and modeling techniques for count data that, to our knowledge, has not been explored and documented to predict spatial distribution and abundance of offshore recreational vessels. Therefore, the objectives of this analysis were to explore the association between offshore recreational vessels and select external variables, as well as to explore different modeling technique to describe mail survey derived count data (i.e., Poisson, negative binomial, hurdle and zero inflated modes, generalized additive models, and generalized mixed models) to find parsimonious models that best describe the seasonal distribution of offshore recreational boating in the study area.

## Methods

### Study area

**Fig 1:**
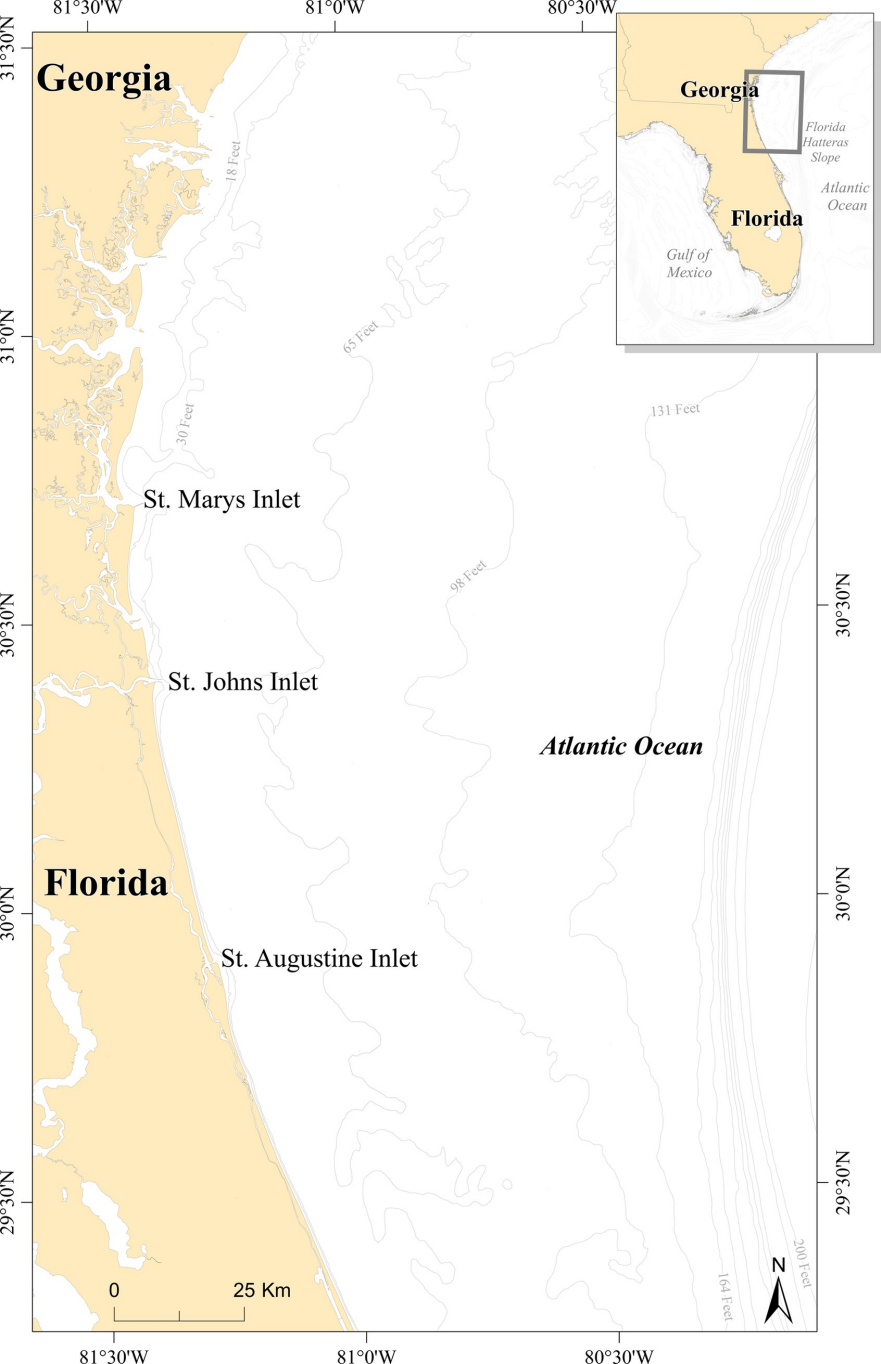
Study area. Northeast Florida (Nassau, Duval, St Johns, Flagler, and Volusia counties) and Southeast Georgia (Camden and Glynn County).

The study area comprised the offshore waters off the coast of Northeast Florida (Nassau, Duval, St Johns, Flagler, and Volusia counties) and Southeast Georgia (Camden and Glynn County) (Fig 1). The study area encompasses 28,523 km^2^, extends 133 km into the Atlantic Ocean, and stretches 233 km along the coasts of Northeast Florida and Southeast Georgia. Several anthropogenic activities occur in the area, including military, commercial, and recreational activities.

### Data collection

#### Recreational boating surveys

A sample of offshore recreational boating routes in the study area was obtained using a map-based questionnaire that was mailed to boat owners between September and October 2012. The mail survey design was based on previous vessel traffic studies [6,13,14,15,16]. The survey instrument and protocol were approved by the Institutional Review Board at the University of Florida. Survey participants received first an invitation/concent letter and survey questionnaire, followed by a reminder post-card (one-week later), and a replacement survey (two-weeks after). Boaters were asked to draw their entire on-the-water route for four boating trips that they had conducted: one each for winter, spring, summer, and fall. The sampling frame consisted of recreational boat owners whose vessels were recorded transiting the navigable inlets in the study area (St Marys, St Johns, and St Augustine) over a period of 17 months (2011–2012). Vessel owner names and addresses were obtained by linking vessel characteristics recorded at the inlets (e.g., registration number or vessel name and hailing port) with information contained in Florida’s and Georgia’s vessel registration systems, as well as the U.S. Coast Guard National Vessel Documentation Center database [23]. Surveys were distributed to all identified recreational boaters (N = 5,034). Completed surveys were digitized into ArcGIS 10.3 [24]. Non-response bias was evaluated by comparing the self-reported age of early (proxy for respondents) and late (proxy for non-respondents) respondents using a *t*-test [25].

Boating routes were digitized as polylines using an equidistant Universal Transverse Mercator (UTM) projection. A sampling grid cell (5.56 x 5.56 km cells) was used to account for disparities in our study area. The dependent variable (number of routes that intersected each grid cell) was calculated using a spatial join of the boating routes and the sampling grid layer. Additionally, we re-coded the dependent variable into a presence/absence matrix by routes and respondents to accommodate for the mixed model analysis of the winter routes.

#### Habitat variables

Several habitat variables were examined to model the relative probability that a recreational vessel will occupy any given grid cell in the study area. Habitat covariates included sea surface temperature (SST), chlorophyll *a* concentrations (Chl), water depth, abundance of artificial reefs, distance from each grid centroid to the nearest inlet (km), and latitude and longitude of each grid centroid.

We created seasonal composite images for chlorophyll *a* concentrations and SST using monthly satellite-derived information from the NASA Ocean Color Webpage [26] obtained by Terra and Aqua MODIS. Only pixels with high quality (quality = 0) were used for SST and chlorophyll *a* concentrations. Water depth data (90 m horizontal resolution) was obtained from the National Centers for Environmental Information [27]. The distance to the nearest inlet was calculated in ArcGIS 10.3 using the centroid of each grid cell and the mouth of each of the inlets in the study area. Information related with artificial reefs was downloaded from the Florida Geographic Data Library [28]. This layer was spatially joined with the sampling grid cells to obtain the number of artificial reefs and/or fish havens in each grid cell.

### Data analysis

#### Habitat model associations

Partial Mantel tests using *Ecodist* [29] in the R software package version 3.0.2 [30] were used to evaluate the association between each of the habitat variables and the abundance of seasonal boating routes. This technique, a non-parametric test based on dissimilarity matrices, considers autocorrelation while assessing the degree of correlation between variables [31,32]. The structure of the dissimilarity matrix is not independent; therefore, we performed significant testing through permutation procedures (1,000 permutations).

#### Density model

The number of recreational boating trips per grid cell is a discrete nonnegative integer or count. There are several modeling techniques used for count data. Regression models for counts are nonlinear with many properties and special features intimately connected to discreteness and nonlinearity [33]. To explore which of those techniques better accommodates our dataset, we tested several modeling techniques: generalized linear models (GLM) with a Poisson distribution, a negative binomial distribution, generalized additive models with negative binomial link (GAM), hurdle negative binomial models, zero-inflated negative binomial models, and a generalized linear mixed effects modeling approach.

The GLM and the GAM consist of three components. The first component corresponds to the distribution of the response variable, the second is the specification of the systematic component in terms of explanatory variables, and the third is the link between the mean and the response variable and the systematic part [34,35]. The Poisson GLM assumes that the mean is equal to the variance. Poisson models consider that count data are always non-negative and tend to be heterogeneous. The negative binomial GLM is an alternative to the Poisson model when there is over dispersion (the variability in the data is greater than the proposed model) relative to a Poisson model [35]. The generalized additive model (GAM) with a negative binomial link was also implemented. GAM is a generalized linear model with a linear predictor involving a sum of smooth functions of covariates, which allows for non-linear relationships between the dependent and the independent variables [34]. To account for the possibility of an excessive number of zeros in the seasonal datasets, the boating trip abundance was also modeled using two-part models. Zero inflation suggest that zeros are derived in two ways. The first are zeros as a result of absence (i.e., the object of interest does not exist in the spatial location) and the second are zeros as a result of non-detection (i.e., the object of interest does exist in the location, but it was not observed). This process results in far more zeros than would be expected for a specific distribution (in our case the negative binomial distribution). It is important to account for zero inflation because it could bias the estimated parameters and lead to over dispersion in the model [35]. In a two-part model, a binomial model is applied where data are considered as zeros versus non-zeros. Then a Poisson or negative binomial model is used to model the non-zero counts. The hurdle negative binomial model is a two-part model that does not discriminate between the origins of the zeros. It assumes that a hurdle should be crossed before values become non-zeros. In this case the distribution of the count part of the model is zero truncated [35]. The zero-inflated negative binomial model (ZINB) is another popular two-part model. It differs from the hurdle model in the sense that it models the zeros as if they are coming from different processes (binomial and count processes). The distribution for the count model in the ZINB is not zero-truncated (the count model is allowed to generate zero values).

Model analyses were conducted in R software package version 3.0.2 [30] using distinct packages. The *MASS* [36] and *car* [37] packages were used for the Poisson and Negative Binomial regression. Hurdle and zero inflated negative binomial models were obtained using the *pscl* package [38]. Generalized additive models were run using *mgcv* [39] and *nlme* [40]. To account for collinearity, a stepwise procedure was implemented and habitat variables with variance inflation factors (VIF) higher than 3 were eliminated from further models [34]. Model diagnostics and autocorrelation estimates for the residuals using Moran’s Index were obtained and model performance was compared using the Akaike Information Criterion (AIC), delta AIC, and AIC weights (using *MuMin*) [41]. Predictions of each model were mapped and compared using regression techniques (major axis regression) on the observed vs. predicted values for each season using *lmodel2* [42,43].

#### GAM model

Based on model performance, the final model analysis was conducted using a GAM approach with a negative binomial distribution and logit link. To minimize overfitting, a penalty term was added to the regression to control for the smoothness of the fitted curve by setting the gamma = 1.4 in the ‘gam’ function to force the effective degrees of freedom to count as 1.4 times the degrees of freedom in the generalized cross-validation score [34]. The smoothness selection was fit using spline-based penalized likelihood estimation. Theta parameters and weighted penalties were determined by AIC, delta, AIC weights, residuals sum of square, and root mean squared deviation estimates. Thin plate regression splines over location were used to account for autocorrelation [44]. We followed a stepwise procedure for model selection. The total deviance explained, the AIC estimates, and the significance of each added variable (*p*>.05) were used to evaluate the role of the independent variables in explaining the observed distribution of recreational vessels. Predictions of each working model were compared with the observed dataset using regression techniques to validate the best model.

#### Mixed effect logistic model (presence/absence)

The generalized mixed model (GLMM) is an extension of the GLM that allows for correlation between the observations through the introduction of random effects [35,45]. Models that include the random effect consider any correlation between survey participants. Mixed effects models feature both fixed and random effects. Fixed effects are unknown or constant variables (observations are independent). If we assume that there is some type of relationship between observations, then those variables are considered random effects [46]. In some cases, survey respondents provided more than one recreational boating trip per season, therefore respondents’ ID was used as a random effect. A model for the winter season was performed in R software version 3.0.2 using the *lme4* package [47]. Pearson correlations, boxplots, and VIF were used to select the habitat variables that were included in the final model. The final model was selected using AIC estimates.

## Results

We had a 19.03% return rate (n = 958 returned surveys). However, we were not able to use all of the returned surveys. Forty-eight percent of those who returned a survey were not our target population (e.g., inshore boaters, boating activities occurred out of our study area) or returned a blank survey. Data collected from 507 surveys were digitized, which yielded a sample of 2,522 boating routes (all four seasons). Difference in the self-reported age for respondents (early) (M = 56.9, SD = 10.8) and non-respondents (late) (M = 55.9; SD = 11.3) was not statistically significant (*t* (340) = 0.86, *p* = 0.39).

The mean count of routes intersecting any grid cell was 9.2 (SD = 20.5). Throughout all seasons, 21% of grid cells showed no counts of recreational vessels. However, this number fluctuated between 13% of grid cells with zero counts in the spring season up to 27% in the winter season.

### Habitat model associations

Seasonal mean SST varied from 19.8°C (SD = 2.2) in winter to 27.3°C (SD = 0.48) in summer. Grid cells that were intercepted by at least one vessel route have a mean SST of 24.02°C (SD = 3.06). Seasonal chlorophyll *a* concentrations ranged from 0.9 mg/m^3^ (SD = 1.07) in spring to 1.29 mg/m^3^ (SD = 1.3) in winter. Vessel routes were recorded at chlorophyll *a* mean concentrations of 1.16 mg/m^3^ (SD = 1.2). Based on *t*-test results, there was a statistically significant difference with respect to the chlorophyll *a* concentration (Table 1). In general, grid cells that were intersected by at least one recreational trip had an average water depth of 38.1 m (SD = 53.9), were closer to inlets (mean distance = 57.0 km, SD = 32.6), and had a higher percentage of artificial reefs (12.4%). When comparing grid cells with recreational trip counts to those without, there is a statistical difference for all environmental variables except for SST (Table 1).

**Table 1:** Descriptive statistics of grid cells associated with the presence and absence of recreational boating routes. Recreational boating routes (Routes), sea surface temperature (SST), chlorophyll *a* concentrations, water depth, distance of the centroid of each grid cell to the nearest inlet (Inlet), and abundance of artificial reefs (Reef).

Variables	Mean (SD)		*t*-test	*p*-value
	Routes = 0	Routes ≥ 1		
Routes	777	2919	na	na
SST (^o^C)	23.8 (3.6)	24.0 (3.1)	-1.6	.11
Chlorophyll a	0.6 (0.8)	1.2 (1.2)	-14.4	< .001
Water depth (m)	-60.6 (91.6)	-38.1 (53.9)	-6.6	< .001
Inlet (km)	87.6 (27.9)	57.0 (32.6)	26.2	< .001
Reef	2.3%	12.4%	-12.2	< .001

Recreational boating trip abundance was significantly correlated year-round with chlorophyll *a* concentration, distance from grid centroids to the nearest inlet, and abundance of artificial reefs. Chlorophyll *a* concentration had the strongest relationship with boating trip abundance (Mantel r = 0.21–0.27), with the strongest correlation observed during the winter season. Pearson’s correlation analysis led to similar conclusions with respect to the relationship between the dependent and independent variables [48].

Simple mantel tests and Pearson’s correlation analysis showed significant associations between SST, chlorophyll *a* concentration, distance to the nearest inlet, and latitude for all seasons. Water depth, SST, and distance to the nearest inlet variables were excluded from further analyses because they showed the highest VIF. Further analyses were conducted using only chlorophyll *a* concentration, latitude and longitude, and abundance of artificial reefs as explanatory variables.

The presence/absence model for the winter season allowed for the incorporation of other independent variables, such as boaters’ age and boating experience, total route length, inlet where the route originated, and vessel length. Distance to the nearest inlet (r = -0.18), total route length (r = 0.1), chlorophyll *a* concentration (r = 0.1), and longitude (r = -0.1) showed some relationship with the presence/absence of recreational boating routes.

### Density model–comparison of count data models

**Fig 2:**
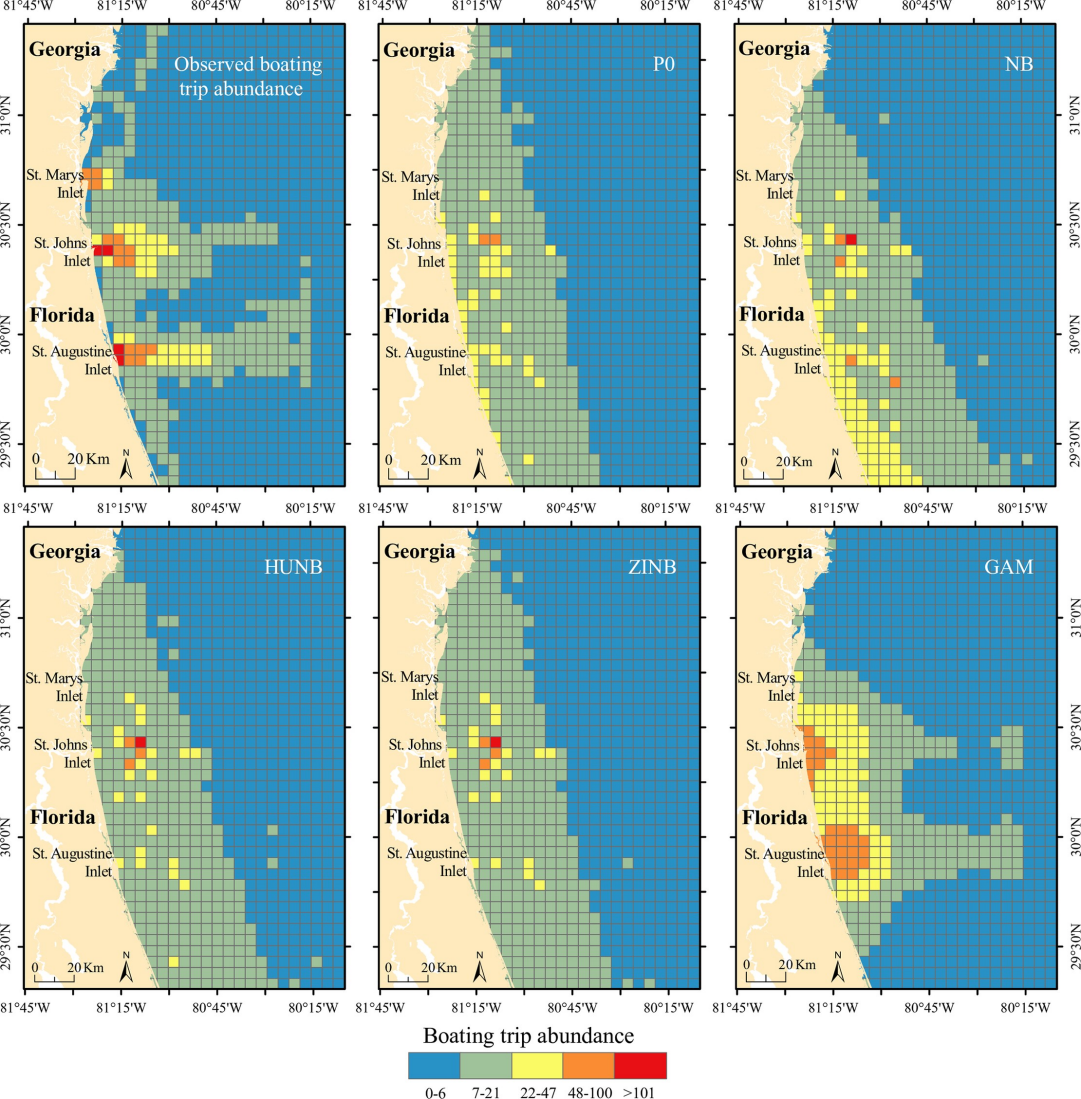
Observed and predicted boating trip abundance generated by different models for the winter season: Poisson model (PO), negative binomial model (NB), hurdle negative binomial model (HUNB), zero inflated negative binomial model (ZINB), and generalized additive model (GAM).

Based on AIC estimates, the generalized additive model (GAM) fit the data better than the other evaluated models (Table 2). Analysis of the residuals also corroborated this. When the normal probability plots of deviance residuals for the different models were compared, only the residuals from the negative binomial and the GAM models did not show any unusual pattern. Furthermore, when the predicted values generated by each of the models for the different seasons were compared with the observed values of the dependent variable (boating trip abundance), it was evident that the model with the best fit was the GAM (Fig 2).

**Table 2:** Model comparison for the count data for different seasons. k = number of model parameters, RSS = residual sum of squares, weights = AIC weights, RMSD = root mean square deviation.

Model	Winter					Spring					Summer					Fall				
	k	RSS	AIC	Delta	Weight	k	RSS	AIC	Delta	Weight	k	RSS	AIC	Delta	Weight	k	RSS	AIC	Delta	Weight
Poisson (PO)	5	7289	9626	5387	<0.001	5	9011	12035	6864	<0.001	5	10855	13352	8942	<0.001	5	7319	9878	5441	<0.001
Negative binomial (NB)	6	981	4714	475	<0.001	6	1000	5573	401	<0.001	6	966	5036	627	<0.001	6	974	4816	380	<0.001
Hurdle (HUNB)	11	960	4639	401	<0.001	9	997	5522	351	<0.001	9	929	5030	620	<0.001	10	1139	4779	343	<0.001
Zero-inflated (ZINB)	11	1024	4554	315	<0.001	9	948	5557	386	<0.001	9	987	4988	579	<0.001	10	1244	4810	373	<0.001
Generalized additive (GAM)	19	477	4239	0	0.99	25	421	5171	0	0.99	19	508	4410	0	0.99	18	569	4437	0	0.98

### GAM model

Model selection (stepwise) was performed for each season. For most seasons, a model that included latitude, longitude, interaction between latitude and longitude, chlorophyll *a* concentration, and abundance of artificial reefs resulted in the best fit (higher explained deviance and lower AIC). The only exception was the model for the summer season, which did not include chlorophyll *a* concentration. The best model explained between 86.1% of the total deviance in the fall and up to 88.6% in summer season (Table 3).

**Table 3:** Model selection to test for the spatial distribution of offshore recreational boating trips for each season. x = longitude, y = latitude, x:y = interaction between longitude and latitude, chl = chlorophyll a concentrations, ref = reef abundance per sampling unit, and s = thin plate regression spline fit, k = number of model parameters, RSS = residual sum of squares, weights = AIC weights, RMSD = root mean square deviation.

Model	Explanatory variables					Model parameters							Observed vs Predicted	
	s(x:y)	s(x)	s(y)	s(chl)	s(ref)	k	RSS	AICc	Delta	Weight	% deviance explained	R-Sq (adj)	R-sq	RMSD
WINTER														
5	+	+	+	+	+	51	683	3644	0	0.99	87.1	0.61	0.64	9.0
4	+	+	+	+		53	696	3661	17	0	86.9	0.61	0.65	9.0
3	+	+	+			47	724	3675	32	0	86.3	0.57	0.61	9.0
2	+	+				36	957	3884	240	0	81.9	0.39	0.41	11.3
1	+					24	1165	4066	422	0	78.0	0.38	0.39	11.5
SPRING														
5	+	+	+	+	+	51	705	4581	0	0.99	86.2	0.78	0.79	7.7
4	+	+	+	+		50	721	4593	13	0.00	85.8	0.76	0.78	8.0
3	+	+	+			44	750	4609	28	0	85.2	0.75	0.76	8.2
2	+	+				35	869	4709	129	0	82.9	0.62	0.63	10.2
1	+					24	1044	4860	279	0	79.4	0.56	0.57	11.0
SUMMER														
6	+	+	+		+	48	843	4057	0	0.91	88.6	0.62	0.64	15.1
5	+	+	+	+	+	45	855	4061	5	0.09	88.4	0.63	0.65	14.9
3	+	+	+			46	883	4090	34	0	88.0	0.61	0.63	15.3
4	+	+	+	+		47	883	4092	66	0	88.0	0.61	0.63	15.4
2	+	+				34	1150	4331	275	0	84.4	0.47	0.49	18.0
1	+					24	1268	4428	371	0	82.8	0.43	0.45	18.7
FALL														
5	+	+	+	+	+	52	753	3978	0	0.99	86.1	0.67	0.71	9.9
4	+	+	+	+		49	772	3990	12	0.00	85.8	0.67	0.71	9.9
3	+	+	+			45	801	4011	33	0	85.2	0.61	0.63	10.8
2	+	+				36	1081	4270	292	0	80.1	0.45	0.47	12.9
1	+					24	1240	4403	425	0	77.2	0.41	0.43	13.4

**Fig 3:**
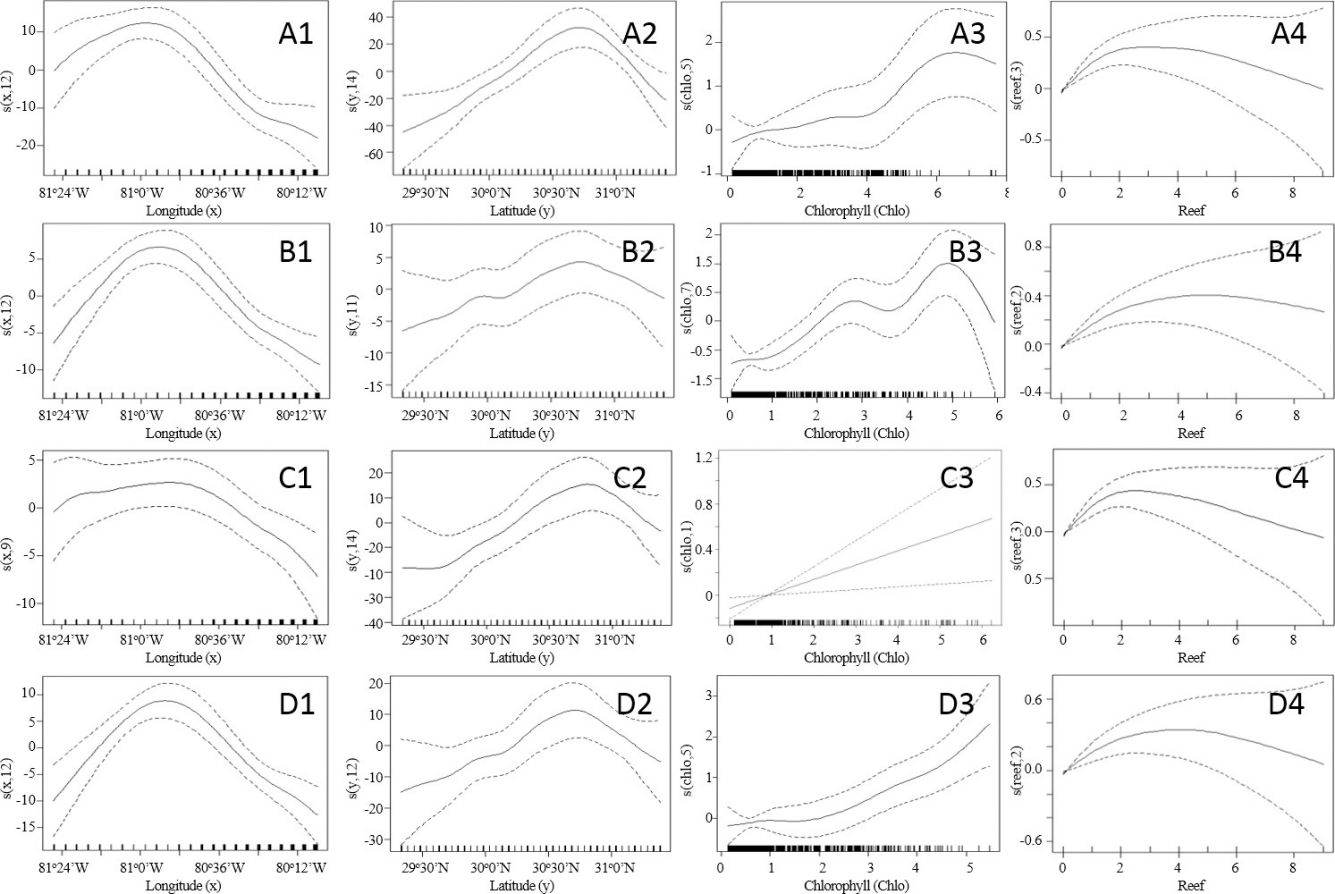
Smoothed curve of the additive effect of covariates modeling the abundance of offshore recreational boating trips in the southeastern United States. Winter (A), Spring (B), Summer (C), and Fall (D). Predictor variables include longitude (x), latitude (y), chlorophyll *a* concentrations (Chlo), and abundance of artificial reefs (Reef). Solid line depicts the estimate of the smooth function and dashed lines indicate 95% confidence bands. Estimated degrees of freedom for each smoothed variable are shown on the y-axis.

Offshore recreational vessel abundance showed a nonlinear relationship with location (longitude and latitude). The distribution of recreational vessels over the longitude (west-east) axis (x) showed an increasing pattern with peaks around the 81^o^0’W and 80^o^48W longitudes (Fig 3). However, this distribution was wider during the summer season followed by the winter season, with vessels travelling further east. Over the summer and fall, the smoothing function showed a narrowed size distribution on the longitude axis (Fig 3). The smoothing functions for the distribution of recreational boating trips for the latitude axis (y) showed an increasing pattern from south to north, up to a latitude of 30^o^45’N (north of St. Marys inlet) (Fig 3).

The smoothing function for chlorophyll *a* concentration in the model varied seasonally. For the winter season, as the concentration of chlorophyll *a* increased so did the abundance of recreational vessels, with a peak between 5–7 mg/m^3^ (Fig 3). During the spring season a higher abundance of vessels occurred between 4.5–5.5 mg/m^3^, followed by another peak between 2.5–3.5 mg/m^3^ (Fig 3). For the summer season, chlorophyll *a* concentration showed a linear relationship with respect to vessel abundance (Fig 3); however, this relationship was not significant, and this variable was dropped from the final summer season model. Over the fall season vessel abundance increased with increasing chlorophyll *a* concentration (Fig 3).

The relationship between the distribution and abundance of offshore recreational vessels and the abundance of artificial reefs was similar for all four seasons. In general, the abundance of vessels increased with increasing numbers of artificial reefs, up to 2–4 reefs per grid cell. However, as the number of artificial reefs increased so did the uncertainty (wider confidence intervals) (Fig 3).

**Fig 4:**
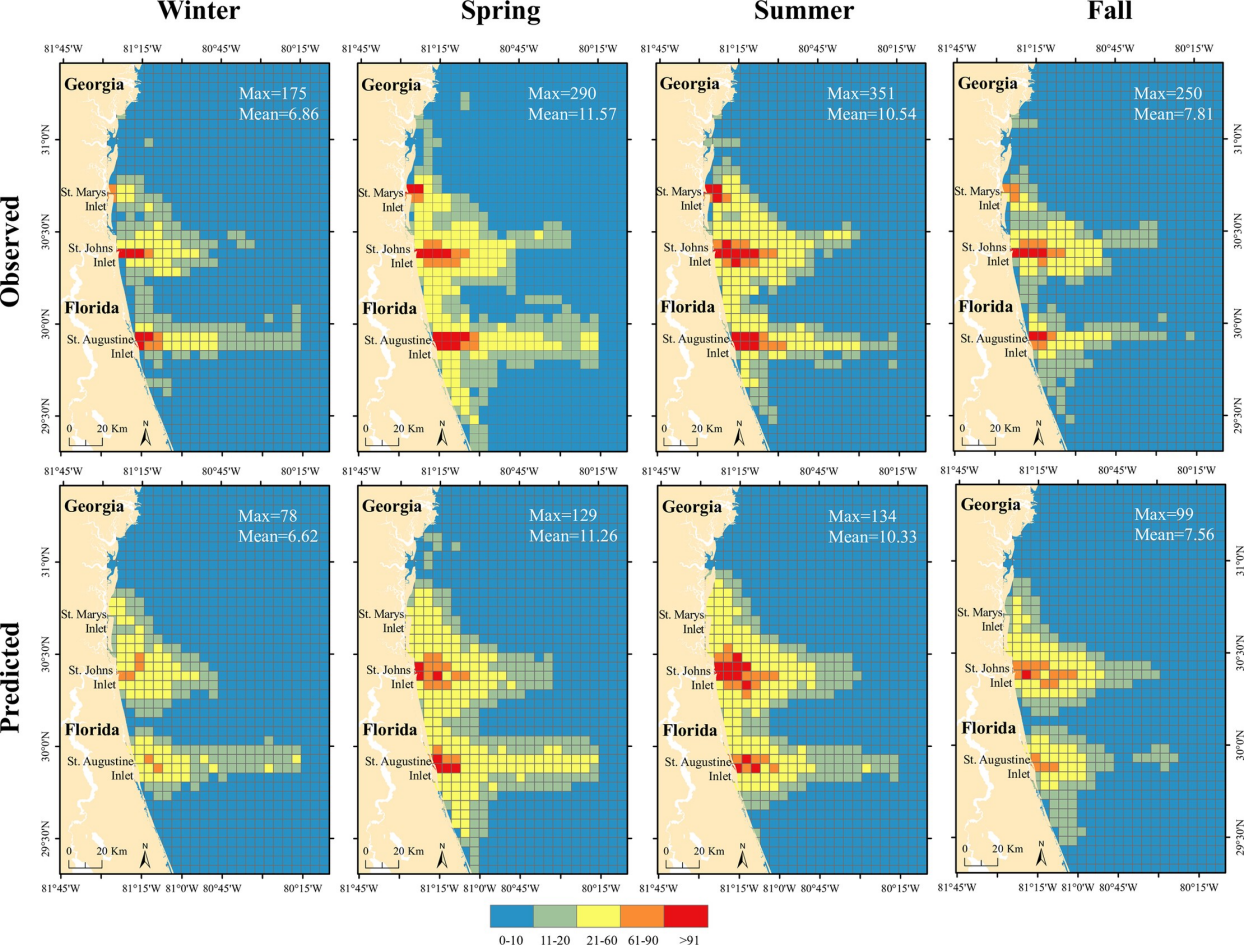
Observed and predicted offshore recreational boating trip abundance per grid cell for different seasons using GAMs.

Model predictions were tested by comparing observed and predicted values. Selected models for each season showed R-square values between 0.64 and 0.79 (Table 3). All models underestimate boating trip abundance at high values, especially for grid cells near inlets. Fig 4 shows the observed and predicted distribution and abundance of recreational vessels. Even though the models show similar means (e.g., observed mean for winter was 6.86 and the mean of the predicted values was 6.62), model predictions did not reach maximum values as extreme as those observed in the training data set. Moran’s I estimates on the residuals did not show any significant sign of autocorrelation (Moran’s I ranged from 0.34, *p*-value >.05 for summer up to 0.37, *p*-value >.05 for the spring season).

### Mixed effect logistic model (GLMM)

**Fig 5:**
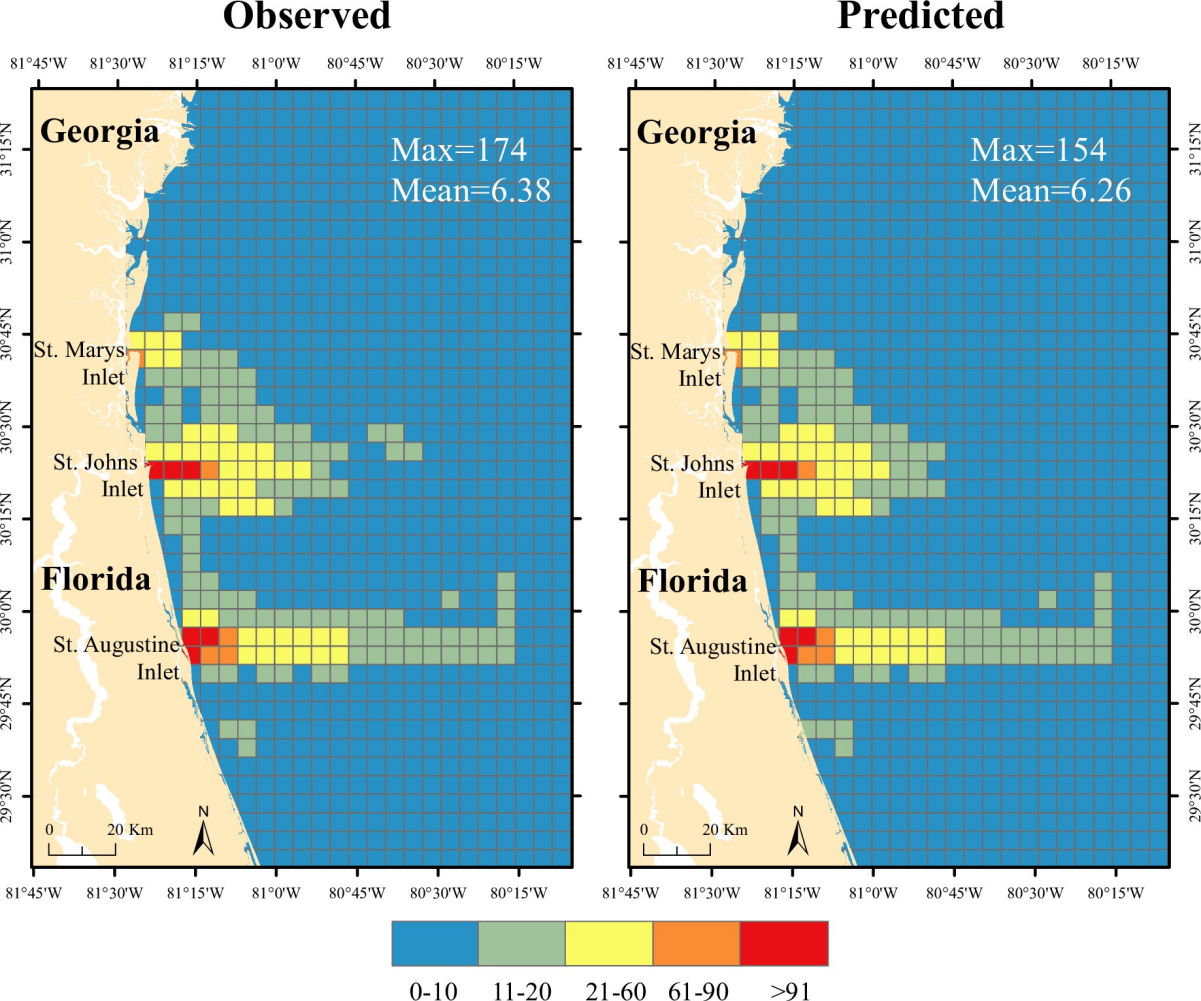
Presence/absence and predicted offshore recreational boating trip abundance for the winter season from the GLMM.

Model selection (stepwise procedure) identified latitude, longitude, and vessel size (length) as the only significant fixed variables while controlling for correlation between the information provided by boaters (boater’s ID). Predictions from the best model for the GLMM are shown in Fig 5. In this case, the cumulative predicted values reached maximum values closer to the observed (cumulative value predicted by GLMM = 154; Max value observed = 174). Areas near the St. Augustine, St. Johns, and St. Marys inlets show the highest predicted values.

## Discussion

Our results show that the seasonal distribution of offshore recreational vessels can be explained mainly by geographical variables such as latitude and longitude, abundance of artificial reefs, and chlorophyll *a* concentrations. Neither SST nor water depth showed any effect on the seasonal distribution of recreational vessels. Other studies have used water depth [18] and SST [7] to describe boat traffic, but the temporal scale in these cases was shorter than our study (e.g., Wu et al., 2009 used hourly, weekly, and monthly estimates), which could explain the differences. Furthermore, SST and water depth were highly correlated with other variables and to avoid problems with multicollinearity they were not included in the final seasonal models. By analyzing the dataset using mixed model methods, we were able to measure the effects of variables related to boaters, their boats, and their self-mapped boating trips. In general, when used to describe presence/absence patterns of boating routes in the sampling grid cells, all the relationships between these variables were weak, ranging from -0.18 to 0.1 (with 1 being highly correlated), Nevertheless, the analyses of the dataset using this methodology allowed us to corroborate the results obtained by the GAM analyses.

The frequency of offshore recreational boating trips per spatial cell follows a negative binomial distribution. Although there are a great number of modeling approaches that can be used to describe and predict offshore recreational boating trips, our results show that the GAM produced a superior model with better fit than did the other studied models. The use of spline functions to describe the relationship between the dependent and independent variables proved to be more effective.

The most parsimonious model for almost all seasons includes latitude and longitude, chlorophyll *a* concentrations, and abundance of artificial reefs. Because there are only a limited number of access points to offshore waters, we expected location (in the form of latitude and longitude) to have an important role in describing the distribution of offshore recreational vessels. Sidman and Fik [20] also highlighted the importance of geographic and network variables in modeling the distribution of recreational vessels and destination choices. Recreational vessel distribution has also been linked with the distribution of target fish species. Fishing is the leading activity of recreational vessels in Florida. Sidman and others [16] found that 73% of recreational boaters in Brevard County (adjacent to our study area) engaged in fishing activities. Several studies found a link between concentrations of chlorophyll a and sea surface temperature and fishery yields [49,50]. In the case of recreational fisheries, Wall and others [51] found that positive catch rates of king mackerel (*Scomberomorus cavalla*) were significantly influenced by chlorophyll a values and temporal variables (season and year). As a result, we included chlorophyll a concentration as a proxy variable for fish distribution (which is the main target of anglers). Overall, we found a positive effect of chlorophyll a concentration on the distribution of recreational boating trips. The only exception for this finding was the boating routes described over the summer months. Consequently, we included chlorophyll *a* concentration as a proxy variable for fish distribution (which are the main target of anglers). In general, we found a positive effect of chlorophyll *a* concentration on the distribution of recreational boating trips. The only exception for this finding was the boating routes described over the summer months. During the summer, chlorophyll *a* concentrations were more homogeneously distributed throughout the study area, resulting in non-significant differences between grid cells with and without boating routes and these variables.

We also included abundance of artificial reefs in our analysis due to their relationship with fish abundance [52,53,54]. Based on our results, there seems to be a positive relationship with abundance of artificial reefs and offshore recreational boating routes for all seasons. Nevertheless, we need to be cautious when interpreting this observed association given that the placement of artificial reefs is likely predicated on several factors (e.g., convenience, near inlets, or close to specific habitats). More research is needed to clarify whether recreational boaters are really targeting areas near artificial reefs or if the observed relationship is an effect of the placement of these artificial structures at vessel transiting areas.

As expected, there were slight differences in the seasonal distribution of recreational vessels, with the spring season showing a more dispersed distribution when compared with other seasons. Adverse climatic conditions (e.g., decreased temperatures, rough sea states) over the winter and fall seasons, and nearshore locations of target fish species may influence how far east (longitudinal movements) recreational boating trips reach; which would affect not only the number of boating trips but also their distribution. With respect to the north-south movements (latitudinal), two areas were shown to be of great importance: St. Johns inlet and St. Augustine inlet. Year-round, most of the boating trips departed from latitudes that correspond with these two inlets. However, we did observe seasonal changes with respect to the abundance of boating trips originating from these inlets. For the winter and spring seasons we observed a greater number and a wider distribution of boating trips at St. Augustine inlet. This inlet is closest (shortest distance) to the Florida Hatteras slope and to some important deep-water fishing areas, such as “The Ledge” (Waterproof Chart #125F). Over the summer and fall seasons, even though the area experiences an increase in temperature and calmer waters, it also experiences an increase in weather events such as hurricanes and tropical storms. During the study period (2011–2012) there were 39 tropical storms and 17 hurricanes [55,56], some of which could have affected the observed distribution of boating routes and ultimately affected predicted values generated from those observations. Our dataset has limitations due to the data collection timeframe and the temporal scale of our study. More on-the-ground data is needed at different temporal and spatial scales to clarify the effect of major weather events on the distribution of offshore recreational boating trips in the study area.

Although our dataset conforms to a negative binomial distribution, there are some high values located near the inlets that affected the predicted values generated for each of the best seasonal models. In most of the instances, those high values could be considered outliers. However, because it was necessary to consider them due to the nature of our study, they were not discarded from our analyses. Nonetheless, when we compared the predictions generated from the GAM models with the presence/absence approach (GLMM), there is a good level of agreement with respect to the predicted values. Therefore, it is important to point out that although our models showed a moderate fit, they present limitations that should be taken into account when using this information for spatial planning and management purposes.

It is important to acknowledge that the findings of our study may be limited to the surveyed sample because the return rate of our mail survey (19%) might be considered low. The main issue with low response rates is that non-respondents may be underrepresented in this sample, limiting the generalizability of the results to the population of interest [57]. However, this is typical of recreational boater surveys, with similar studies reporting return rates between 19–23% [13,14,15,16].

### Management implications

The information provided by this study can be used by managers and law enforcement to determine areas with high/low probability of recreational vessel occurrence and abundance. The study area is large, and this information could help prioritize areas for spatial planning and management purposes. The study area is also very complex, with a number of maritime activities and several marine species co-occurring. Therefore, these models could provide information to analyze the distribution and overlap of recreational boating trips with other maritime activities (e.g., cargo ships, commercial vessels) and species (e.g., right whales, sea turtles, sharks) that utilize this area. These analyses could be used to decrease harmful interactions among these groups and activities.

## Supporting information

S1 File Map-Based_Survey. Questionnaire used to survey offshore recreational boaters in the southeastern United States.
(PDF)

S2 File Offshore recreational vessels. Abundance of offshore recreational vessels (vessels) and co-variables used to model offshore recreational trips in the southeastern United States. Co-variables are: longitude (x), latitude (y), sea surface temperature (sst), chlorophyll a concentration (chlo), water depth (bathy), abundance of artificial reefs (reef), distance to the nearest inlet (inlet), and season.
(CSV)
