# Supplementary material for: Modeling the spatial and seasonal distribution of offshore recreational vessels in the southeast United States
Source: PLoS One. 2018 Nov 28;13(11):e0208126. doi: 10.1371/journal.pone.0208126 (PMC6261638; doi:10.1371/journal.pone.0208126)
Supplement: S1 File — Questionnaire used to survey offshore recreational boaters in the southeastern United States. (PDF) [file pone.0208126.s001.pdf]

UF | UNIVERSITY of FLORIDA

Florida Sea Grant College Program
Boating and Waterway Planning Program

Bldg. 107 Mowry Road
PO Box 110760
Gainesville, FL 32611-0760
(352) 392-6234
fas-boat@ifas.ufl.edu

Studying Recreational Boating Patterns in Northeast Florida
A Survey Conducted by the University of Florida
Sea Grant College Program with funds from NOAA

Dear Boat Owner / Operator,

We are asking you to participate in a boating study to characterize recreational boating patterns in the near- and off-shore waters of Northeast Florida. A goal of the study is to ensure that decisions about the siting of boating infrastructure and resource protection, including right whales, are based on information that includes input from the boating community. Please help in this effort by completing our questionnaire. Florida Sea Grant has worked with coastal communities throughout the state to enhance boating infrastructure, improve navigation access to local waterways, and preserve working waterfronts.

You are one of a small number of recreational boaters selected to receive this survey, so your response is very important. Please rest assured that your responses to this survey will remain strictly confidential. Answers will NOT be linked to individuals. Your name and address will NOT be made available to anyone. The survey number on the questionnaire is only to track returns and to enable us to send a copy of the Northeast Florida map used in this survey to those who request one.

If you have any questions about the study you may contact the project's principal investigator, Dr. Bob Swett, (352) 392-6234, or by email fas-boat@ifas.ufl.edu. If you would like more information about your rights as a survey participant, feel free to contact the University of Florida Institutional Review Board, PO Box 112250, Gainesville, FL 32611, (352) 392-0433.

We are most grateful for your help in this project.

Robert A. Swett
Bob Swett, Ph.D.
Associate Professor
University of Florida

Part 1.

FOR EACH OF THE FOUR SEASONS – WINTER, SPRING, SUMMER, AND FALL – PLEASE DRAW A ROUTE YOU TOOK WHEN BOATING OFFSHORE IN NORTHEAST FLORIDA

"Offshore" refers to waters off the coast that are accessed through an inlet.

Please use the map provided to draw the routes of offshore boating trips that you took during the **most recent** winter (DEC, JAN, FEB), spring (MAR, APR, MAY), summer (JUN, JUL, AUG), and fall (SEP, OCT, NOV) seasons. For example, if you boated offshore during all four seasons, then you will draw four routes—one for each season. However, if you boated offshore during only one of the seasons, you will draw one route on the map.

Please refer to the box labeled "INSTRUCTIONS FOR DRAWING ROUTES" found below and on the map to complete this part of the questionnaire. Thank you.

INSTRUCTIONS FOR DRAWING ROUTES

Please draw the entire ON-THE-WATER route for each season, as accurately as you can.

- Draw a solid line to represent the trip for EACH SEASON. Use arrowheads to indicate direction of travel.

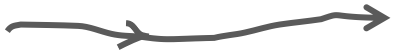

- Please identify each route with the SEASON that it represents, as illustrated below:

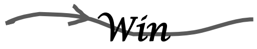

WINTER (December, January, and February)

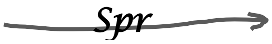

SPRING (March, April, and May)

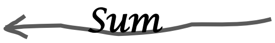

SUMMER (June, July, and August)

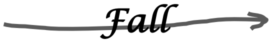

FALL (September, October, and November)

- If you followed the same route for different seasons, just identify both seasons on the route:

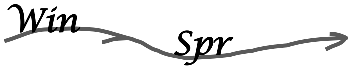

- Mark the START of each route with the letter "S" and the END with the letter "E".
- Indicate a round trip that uses the same route out and back with arrowheads in both directions:

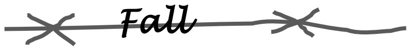

- Mark destinations along your routes with the letter "D".
- If a trip continued beyond the boundary of this map, draw the route to the boundary and indicate the final destination (reef, buoy, fish haven, geographic coordinates, etc) at the map's edge.

PART 2. QUESTIONS ABOUT THE ROUTES YOU DREW ON THE MAP

QUESTION 1. About what time did each trip that you drew on the map begin and end? (*for example, 7:30AM and 1:30PM*). If a trip lasted more than one day (*overnight trips*) please write in the total number of days.

	AT WHAT TIME DID YOUR BOATING TRIP BEGIN?	AT WHAT TIME DID YOUR BOATING TRIP END?	IF IT WAS AN OVERNIGHT TRIP, HOW MANY DAYS DID THE TRIP LAST?
WINTER TRIP			
SPRING TRIP			
SUMMER TRIP			
FALL TRIP			

QUESTION 2. For each trip that you drew on the map, what was the average amount of time (in hours and minutes) that you spent at the destinations you marked on the routes? (*for example, 2 hours and 30 minutes*)

	AVERAGE AMOUNT OF TIME SPENT AT DESTINATIONS	
	HOURS	MINUTES
WINTER TRIP		
SPRING TRIP		
SUMMER TRIP		
FALL TRIP		

QUESTION 3. Please check the day of the week that you took each of the trips that you drew on the map. If you drew an overnight trip, please check the day of the week that the trip began.

	MON	TUE	WED	THU	FRI	SAT	SUN
WINTER TRIP	❑	❑	❑	❑	❑	❑	❑
SPRING TRIP	❑	❑	❑	❑	❑	❑	❑
SUMMER TRIP	❑	❑	❑	❑	❑	❑	❑
FALL TRIP	❑	❑	❑	❑	❑	❑	❑

QUESTION 4. Please indicate the departure site type that you used for each trip that you drew on the map.

	BOAT RAMP	MARINA WET SLIP	MARINA DRY SLIP	HOME OR CONDOMINIUM DOCK	SHORELINE OR CAUSEWAY
WINTER TRIP	❑	❑	❑	❑	❑
SPRING TRIP	❑	❑	❑	❑	❑
SUMMER TRIP	❑	❑	❑	❑	❑
FALL TRIP	❑	❑	❑	❑	❑

QUESTION 5. Please check the box that best describes the type of boat that you used for each trip that you drew on the map.

BOAT TYPE	WINTER TRIP	SPRING TRIP	SUMMER TRIP	FALL TRIP
Sailboat	❑	❑	❑	❑
Speed Boat (runabout/bowrider)	❑	❑	❑	❑
Open Fisherman (Flats/Skiff/Center console)	❑	❑	❑	❑
Offshore Sportfisherman	❑	❑	❑	❑
Power Cruiser	❑	❑	❑	❑
Other (specify):	❑	❑	❑	❑

QUESTION 6. Please write the length (in feet) of the boat that you used for each trip that you drew on the map.

	BOAT LENGTH (FEET)
WINTER TRIP	
SPRING TRIP	
SUMMER TRIP	
FALL TRIP	

QUESTION 7. Please write the number of days that you operated your boat offshore in Northeast Florida during each of the most recent seasons.

	WINTER SEASON (DEC, JAN, FEB)	SPRING SEASON (MAR, APR, MAY)	SUMMER SEASON (JUN, JUL, AUG)	FALL SEASON (SEP, OCT, NOV)
Number of offshore boating trips				

IF YOU DEPARTED FROM A RAMP, MARINA, OR SHORELINE/CAUSEWAY, PLEASE GO TO PART 3 ON PAGE 4.

IF YOU DEPARTED FROM A HOME OR CONDOMINIUM DOCK, PLEASE GO TO PART 4 ON PAGE 5.

PART 3. QUESTIONS ABOUT YOUR DEPARTURE SITES

QUESTION 8. Please write below the name or location of the ramp, marina, dry stack, or shoreline/causeway that you used for each of the trips that you drew on the map. (*See map for reference*.)

	NAME OR LOCATION OF THE DEPARTURE SITE TYPE
WINTER TRIP	
SPRING TRIP	
SUMMER TRIP	
FALL TRIP	

QUESTION 9. How important to you is each of the following factors when selecting the ramp, dry stack, marina, or shoreline that you use when boating offshore in Northeast Florida? (*In the table below, check the box that best describes how important each factor is to you*.)

FACTORS	IMPORTANT	SOMEWHAT IMPORTANT	NEUTRAL	SOMEWHAT UNIMPORTANT	UNIMPORTANT
Close to an inlet (near offshore waters)	❑	❑	❑	❑	❑
No launching or parking fees	❑	❑	❑	❑	❑
Restroom availability	❑	❑	❑	❑	❑
Adequate parking	❑	❑	❑	❑	❑
Close to my home (shorter drive time)	❑	❑	❑	❑	❑
Nearby amenities (store, restaurant, hotel, etc.)	❑	❑	❑	❑	❑
Safe and secure parking area	❑	❑	❑	❑	❑
Close to my favorite boating spots	❑	❑	❑	❑	❑
Availability of fishing supplies, bait	❑	❑	❑	❑	❑
Hours of operation	❑	❑	❑	❑	❑
Gas, pump-out, or maintenance service	❑	❑	❑	❑	❑
Other (specify):	❑	❑	❑	❑	❑

QUESTION 10. Suppose that a new ramp, marina, or dry stack were built and it had all the amenities you could want and solved problems you now experience. What is the maximum on-the-water distance that you would travel from that facility to the nearest inlet (the distance beyond which you would not use the facility)?

On the hypothetical diagram below of a navigable river leading to an inlet, please circle the on-the-water distance upstream from the inlet that represents the maximum distance that you would be willing to travel between the facility and the inlet.

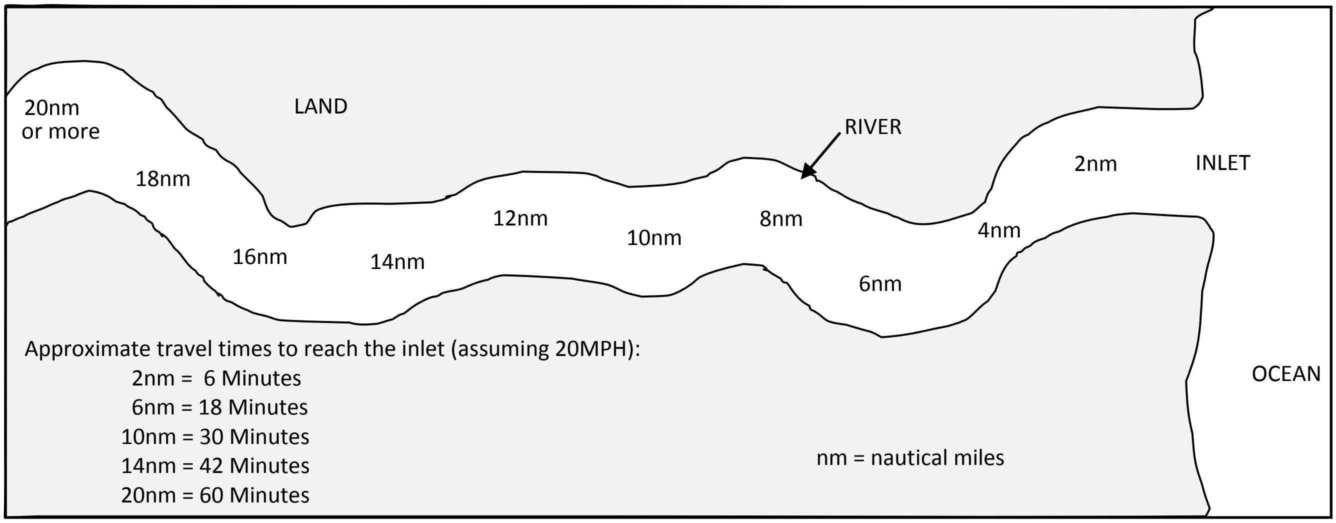

PART 4. PLEASE DESCRIBE YOURSELF

QUESTION 11. How many years have you boated offshore in Northeast Florida?_____________ (*Years*)

QUESTION 12. Have you ever completed a boat safety or seamanship course? ❑ Yes ❑ No

QUESTION 13. In what year were you born? ________________

QUESTION 14. What is the ZIP code of your primary Florida residence?_______________

QUESTION 15. Please check this box ❑ if you would like us to mail you a duplicate copy of the survey map.

QUESTION 16. Please provide any other comments that you would like us to know about your boating trips, boating facilities that you use, waterway improvements, or information/actions that would increase your boating enjoyment or help care for your boating environment.

COMMENTS:

PLEASE RETURN THE QUESTIONNAIRE AND MAP IN THE ENCLOSED POSTAGE-PAID ENVELOPE.
THANK YOU VERY MUCH FOR YOUR TIME AND PARTICIPATION!

QUESTIONNAIRE CONTROL NUMBER
(Used only to keep track of survey returns so that we do not bother you with reminders, and to enable us to send a copy of the survey map to those who request one.)

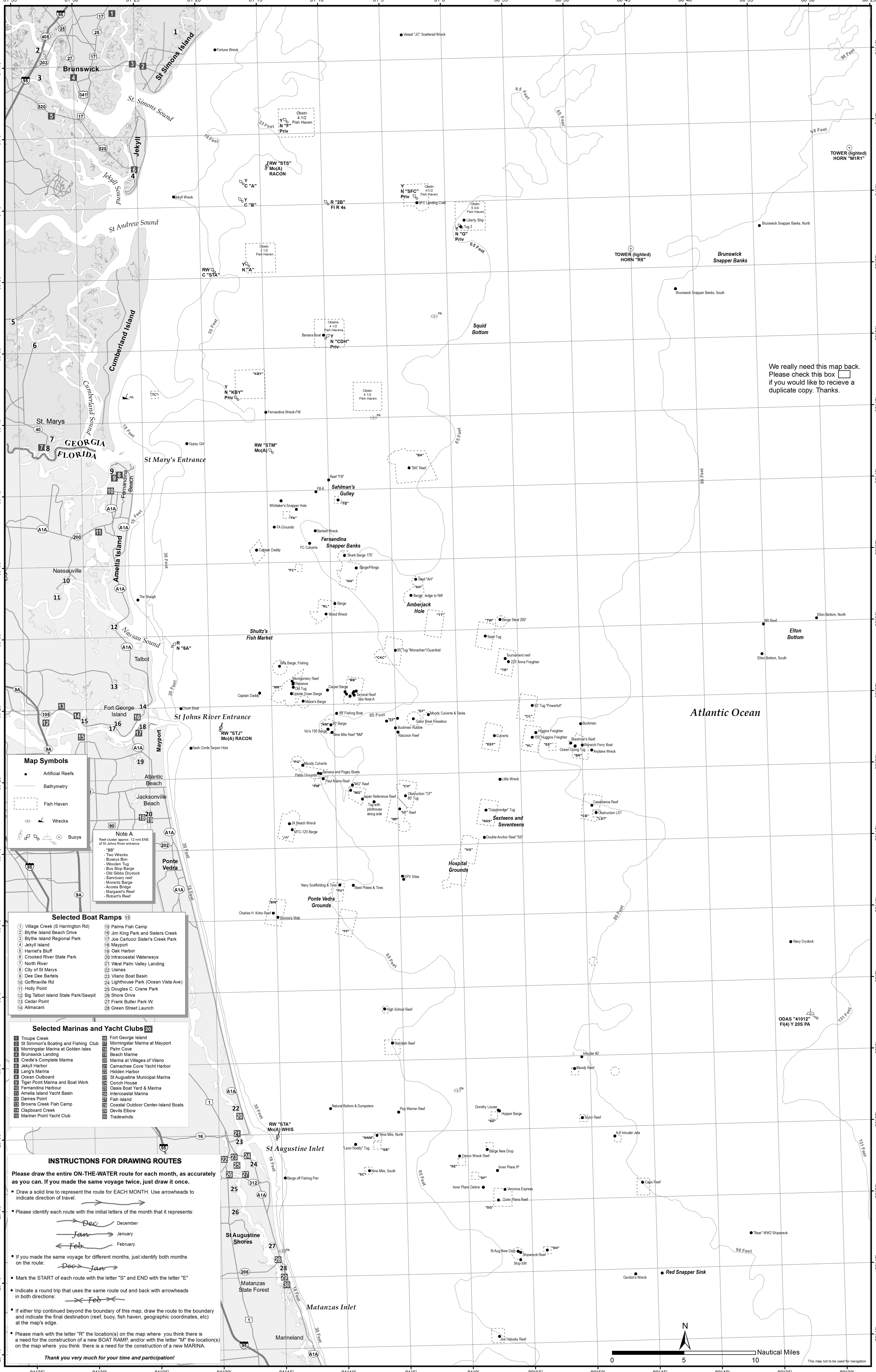
Map Symbols
Artificial Reefs
Bathymetry
Fish Haven
Wrecks
Buoys
Note A
Reef cluster approx. 12 nmi ENE of St Johns River entrance
- "BB"
- Two Wrecks
- Buseys Bon
- Wooden Tug
- Bus Stop Barge
- Old Gibbs Drydock
- Sanctuary reef
- Morantz Barge
- Acosta Bridge
- Margaret's Reef
- Robert's Reef
Selected Boat Ramps 15
1 Village Creek (S Harrington Rd)
2 Blythe Island Beach Drive
3 Blythe Island Regional Park
4 Jekyll Island
5 Harriet's Bluff
6 Crooked River State Park
7 North River
8 City of St Marys
9 Dee Dee Bartels
10 Goffinsville Rd
11 Holly Point
12 Big Talbot Island State Park/Sawpit
13 Cedar Point
14 Alimacani
15 Palms Fish Camp
16 Jim King Park and Sisters Creek
17 Joe Carlucci Sister's Creek Park
18 Mayport
19 Oak Harbor
20 Intracoastal Waterways
21 West Palm Valley Landing
22 Usinas
23 Vilano Boat Basin
24 Lighthouse Park (Ocean Vista Ave)
25 Douglas C. Crane Park
26 Shore Drive
27 Frank Butler Park W.
28 Green Street Launch
Selected Marinas and Yacht Clubs 20
1 Troupe Creek
2 St Simmon's Boating and Fishing Club
3 Morningstar Marina at Golden Isles
4 Brunswick Landing
5 Credle's Complete Marina
6 Jekyll Harbor
7 Lang's Marina
8 Ocean Outboard
9 Tiger Point Marina and Boat Work
10 Fernandina Harbour
11 Amelia Island Yacht Basin
12 Dames Point
13 Browns Creek Fish Camp
14 Clapboard Creek
15 Mariner Point Yacht Club
16 Fort George Island
17 Morningstar Marina at Mayport
18 Palm Cove
19 Beach Marine
20 Marina at Villages of Vilano
21 Camachee Cove Yacht Harbor
22 Hidden Harbor
23 St Augustine Municipal Marina
24 Conch House
25 Oasis Boat Yard & Marina
26 Intercoastal Marina
27 Fish Island
28 Coastal Outdoor Center-Island Boats
29 Devils Elbow
30 Tradewinds
INSTRUCTIONS FOR DRAWING ROUTES
Please draw the entire ON-THE-WATER route for each month, as accurately as you can. If you made the same voyage twice, just draw it once.
• Draw a solid line to represent the route for EACH MONTH. Use arrowheads to indicate direction of travel.
• Please identify each route with the initial letters of the month that it represents:
Dec December
Jan January
Feb February
• If you made the same voyage for different months, just identify both months on the route:
Dec Jan
• Mark the START of each route with the letter "S" and END with the letter "E"
• Indicate a round trip that uses the same route out and back with arrowheads in both directions:
Feb
• If either trip continued beyond the boundary of this map, draw the route to the boundary and indicate the final destination (reef, buoy, fish haven, geographic coordinates, etc) at the map's edge.
• Please mark with the letter "R" the location(s) on the map where you think there is a need for the construction of a new BOAT RAMP, and/or with the letter "M" the location(s) on the map where you think there is a need for the construction of a new MARINA.
Thank you very much for your time and participation!
We really need this map back. Please check this box if you would like to recieve a duplicate copy. Thanks.
Atlantic Ocean
Brunswick
St Simons Island
Jekyll
Cumberland Island
St. Marys
GEORGIA
FLORIDA
Amelia Island
Nassauville
Fernandina Beach
Talbot
Fort George Island
Mayport
Atlantic Beach
Jacksonville Beach
Ponte Vedra
St Augustine Shores
Matanzas State Forest
Marineland
St Mary's Entrance
St Johns River Entrance
St Augustine Inlet
Matanzas Inlet
Nautical Miles
0
5
10
This map not to be used for navigation
